# The Role of Combined Penetration Enhancers in Nasal Microspheres on In Vivo Drug Bioavailability

**DOI:** 10.3390/pharmaceutics10040206

**Published:** 2018-10-26

**Authors:** Giovanna Rassu, Luca Ferraro, Barbara Pavan, Paolo Giunchedi, Elisabetta Gavini, Alessandro Dalpiaz

**Affiliations:** 1 Department of Chemistry and Pharmacy, University of Sassari, via Muroni 23/a, 07100 Sassari, Italy; grassu@uniss.it (G.R.); pgiunc@uniss.it (P.G.); 2 Department of Life Sciences and Biotechnology, University of Ferrara, via Borsari 46, 44121 Ferrara, Italy; frl@unife.it; 3 Department of Biomedical and Specialist Surgical Sciences, University of Ferrara, via Borsari 46, 44121 Ferrara, Italy; pvnbbr@unife.it; 4 Department of Chemical and Pharmaceutical Sciences, University of Ferrara, via Fossato di Mortara 19, 44121 Ferrara, Italy; dla@unife.it

**Keywords:** combined microsphere, chitosan, cyclodextrin, nasal delivery, nose to brain transport, penetration enhancer, nasal formulation, in vivo studies

## Abstract

Microspheres based on both methyl-β-cyclodextrins and chitosan were prepared by spray-drying as nasal formulations of a model polar drug to analyze, firstly, how the composition of the carrier affects drug permeation across synthetic membranes and, secondly, how it induces systemic or brain delivery of the drug. Microparticles with different weight ratios of the two penetration enhancers (10–90, 50–50, 90–10) were characterized with respect to morphology, size, structural composition, water uptake, and the in vitro drug permeation profile. The leader formulation (weight ratio of 50–50) was then nasally administered to rats; systemic and cerebrospinal fluid (CSF) drug concentrations were analyzed by high performance liquid chromatography (HPLC) over time. Microspheres obtained with a single enhancer, methyl-β-cyclodextrins or chitosan, were administered in vivo as a comparison. The in vitro properties of combined microspheres appeared modified with regard to the polymeric matrix ratio. In vivo results suggest that the optimal drug distribution between CSF and bloodstream can be easily obtained by varying the amount of these two penetration enhancers studied in the matrix of nasal microspheres.

## 1. Introduction

Absorption enhancers are agents included in formulations whose function is to increase absorption of a pharmacologically active drug by enhancing membrane permeation [1]. They are frequently employed in nasal formulations [2,3,4,5] in order to improve drug flux through nasal epithelia [6]. The main mechanisms involved in enhanced permeability of nasal membranes include (i) the transient opening of the tight junction between adjacent cells that favor paracellular diffusion through the intercellular space, and/or (ii) perturbation of lipid bilayer integrity and increased membrane fluidity promoting the transcellular permeation of drugs [1]. Permeation-enhancing excipients are investigated to develop formulations for hydrophilic drugs that have poor nasal membrane permeability [7]; among these, cyclodextrins (CDs) and chitosan are characterized by high biocompatibility, since they are able to induce their enhancing effects without causing damage to nasal mucosa [7].

Cyclodextrins, especially methylated-β-cyclodextrins, have proven to be excellent solubilizers and absorption enhancers in nasal drug delivery [2,8,9,10]. Owing to their ability to remove cholesterol and phospholipids from the outer half of the membrane bilayer, CDs can increase membrane fluidity [11]. Furthermore, methylated-β-cyclodextrins cause an increase in the paracellular permeability of epithelial cell layers because the cholesterol depletion influences the distribution of specific tight junction proteins (claudin 3, claudin 4, and occludin) [12]. Recently, Fenyvesi and co-workers [13] have shown that fluid-phase endocytosis of CDs can contribute to overcoming the mucosal barrier for poorly absorbed drugs.

Chitosan is one of the most-used penetration enhancers in nasal formulations [14,15,16,17]. A combination of mucoadhesion and transient opening of the tight junctions in the cell membrane allows polar drugs to pass by a paracellular pathway [2]. There are a number of chitosan salts that have better characteristics than chitosan itself; these include solubility, mucoadhesiveness, and penetration enhancement ability. The chitosan chloride salt form is the only kind of chitosan that has a specific monograph in the European Pharmacopoeia 8.0 [18].

In addition to high stability, powder formulations can also have an absorption promoting effect [2,17] by exerting a direct effect on the mucosa. Microspheres absorb water from mucus causing dehydration of the epithelial cells which leads to the opening of the tight junctions [19]. For these reasons, the formulation of solid microparticles in the presence of promotion enhancers appears advantageous for nose to brain delivery of drugs. 

Traditionally cyclodextrin and chitosan have been used separately in nasal powder formulation [15,16,20] and no combination has been evaluated. Bshara and co-workers [21] formulated hydroxylpropyl-β-CD and chitosan aspartate in microemulsion and observed that the highest C_max_ and AUC values among all the formulations they tested (formulation without hydroxylpropyl-β-CD and chitosan aspartate and with chitosan aspartate alone) were most likely due to a synergistic effect of both materials on the intranasal permeation of drug. 

Therefore, the aim of this work was to formulate methyl-β-cyclodextrins and chitosan in combination in spray-dried microspheres for nasal administration of a polar drug. In particular, the effects of the microparticles’ composition on drug permeation across synthetic membranes were analyzed, and, then, related to systemic or brain delivery of the drug after intranasal administration. 

Indeed, when administered nasally, a drug can be deposited on the respiratory or olfactory epithelia. In the first case, it may cross the respiratory mucosa by paracellular or transcellular transport, reaching the lamina propria and then blood vessel (systemic circulation) or peripheral trigeminal nerve (brainstem). If, on the other hand, the drug is deposited on the olfactory epithelium, transport to the central nervous system may be either paracellular through olfactory neurones, or transcellular through olfactory epithelial cells [7,22].

N^6^-cyclopentyladenosine was selected as a model polar drug with low molecular weight.

## 2. Materials and Methods 

### 2.1. Materials

Methyl-β-cyclodextrin, Cavasol^®^ W7MPharma (Mw: 1300 g/mol; molecular substitution: 1.7) was purchased from Wacker-Chemie GmbH (München, Germany). Chitosan chloride, Protasan UP CL 113 (Mw: 160,000 g/mol; deacetylation degree, 86%) were purchased from NovaMatrix/FMC Biopolymer (Sandvika, Norway). Tegiloxan3™ silicon oil was kindly gift from Goldschmidth (Essen, Germany). N6-cyclopentyladenosine (CPA) (Mw: 335.36), N6-cyclohexyladenosine CHA, sodium acetate and acetic acid glacial were obtained from Sigma-Aldrich (Milan, Italy). Dimethylsulfoxide (DMSO) was obtained from Merck (Darmstadt, Germany). Methanol, acetonitrile, ethyl acetate, and water high performance liquid chromatography (HPLC) grade were purchased from Sigma-Aldrich (Milan, Italy). Male Wistar rats were purchased from Charles-River, Milan, Italy.

### 2.2. Preparation of Microspheres

Formulations of microspheres were prepared by spray drying technique with a Mini Büchi B-191 spray dryer (Büchi Laboratoriums-Technik AG, Flawil, Switzerland) using methyl-β-cyclodextrin and chitosan as excipients in different percentage ratios chosen on the basis of preliminary studies. Drug to polymer ratio 1/20 was chosen which guarantees an optimal dose for in vivo test and administration of powder amount suitable for rat nasal cavity [23]. The feed solution concentration was 0.5% (*w*/*v*). The microparticles containing both excipients in different weight ratio (10–90, 50–50, and 90–10) were prepared dissolving separately methyl-β-cyclodextrin and chitosan in water, under magnetic stirring, and solutions were mixed before the addition of CPA. The following operating conditions were utilized during spray drying: inlet and outlet temperature, 110 °C and 77 ± 2 °C, respectively; spray flow rate, 500 L/h; pump setting, 5% (1.74 ± 0.09 mL/min); and aspirator setting, 98% regardless solutions composition. The dry particles were put in a desiccator for at least 24 h. Unloaded microspheres based on methyl-β-cyclodextrin (MCb) and loaded microspheres based on chitosan (CH100) or methyl-β-cyclodextrin (MC100) were prepared as comparison.

The codes and composition of the microspheres are presented in Table 1. 

The yields of production were calculated as the weight percentage of the final product after drying, with respect to initial total amounts of drug and excipients used for the preparation.

### 2.3. Microsphere Characterization

#### 2.3.1. Determination of Drug Content and Encapsulation Efficiency 

To determine the real amount of CPA loaded in microspheres, 0.5 mL of water was added to an exact amount of combined formulations (containing about 0.5 mg of drug); then, 45.5 mL of methanol were added and stirred until solution was obtained. Finally, spectrophotometric analysis (UV-160A UV–Visible Recording Spectrophotometer, Shimadzu, Tokyo, Japan) was performed at 270 nm and the drug in solutions was quantified by using the calibration curve obtained in concentrations range from 2.8 to 22.4 mg/L (*y* = 0.067*x* + 0.027, *R*^2^ = 0.9997). Analyses were carried out in triplicate.

Furthermore, an exact amount of MC100 (corresponding to 0.5 mg of CPA) was dissolved in 50 mL of methanol and solution spectrophotometrically analyzed. 

The drug loaded in microspheres (DC) was calculated as the experimentally detected amount of CPA in microspheres and expressed as percentage; encapsulation efficiency (EE), as percentage, was also calculated. The following equations were applied:

$$\mathrm{DC}\left(\%\right)=\left(\frac{\mathrm{rCPA}}{\mathrm{tCPA}}\right)\times \mathrm{tDC}$$

$$\mathrm{EE}\left(\%\right)=\left(\frac{\mathrm{DC}}{\mathrm{tDC}}\right)\times 100$$

where rCPA is the detected amount of CPA in microspheres, tCPA is the drug amount solubilized in feed solution and tDC the percentage of the expected theoretical value.

#### 2.3.2. Morphological Analysis

The morphology of the microspheres was determined by observation on a scanning electron microscope (VP-SEM; Zeiss EVO40XVP, Arese, Milan, Italy). The samples were placed on double-sided tape that had previously been secured to aluminum stubs and then analyzed under an argon atmosphere at an 18 kV acceleration voltage after gold sputtering.

#### 2.3.3. Particle Size Analysis

The particle size and particle size distribution of the microspheres were determined by laser diffraction using a Coulter Laser Sizer LS100Q (Coulter LS 100Q Laser sizer, Beckman Coulter, Miami, FL, USA). All analyses were performed at room temperature. Two mg of microspheres were suspended in 1 mL of Tegiloxan by sonication (about 4–6 s) and then analyzed. The results reported are the averages of triplicate averages. The average particle size of the microspheres was reported as the mean volume-surface diameter, d_vs_ (μm). To quantify distribution width, SPAN was calculated as previously reported [24].

#### 2.3.4. Powder X-ray Diffraction

Powder diffraction spectra were recorded, at room temperature, on a Bruker D-8 Advance diffractometer (Brucker, Karlsruhe, Germany) with graphite monochromatized Cu Kα radiation (λ = 1.5406 Å). The data were recorded at 2θ steps of 0.02° from 3° to 50° with 2 s/step. 

#### 2.3.5. Water Uptake 

The investigation of water uptake capability was carried out by the modified Enslin apparatus as previously described [25]. Microspheres (10 mg) were uniformly dispersed on a cellulose filter disk saturated with MilliQ water, lying on the top of a fritted glass support, connected with a graduated capillary. The volume of water absorbed (μL) along the time (from 0.25 up to 60 min) was measured. The result obtained is the average of three determinations (n = 3; ±SD).

#### 2.3.6. In Vitro Permeation Test of Microspheres

The in vitro drug permeation across synthetic membrane was performed using a modified Franz diffusion system incorporating three in-line flow-through diffusion cells [26]. Regenerated cellulose membranes (pore size 0.45 μm, 47 mm diameter, Sartorius, Goettingen, Germany) saturated with octanol were chosen as lipophilic layer [20]. An amount of microspheres containing about 1.0 mg of CPA was uniformly spread out above the membrane. Acceptor compartment was filled with 100 mL of pH 6.5 phosphate buffer at 37 ± 0.5 °C, to guarantee sink condition. The flux of the medium was 6 mL/min. Permeation system was automatically coupled to UV-spectrophotometer (UV-1800, UV–Visible Recording Spectrophotometer, Shimadzu, Tokyo, Japan) and the fluid was analyzed at 270 nm at predetermined times (from 30 up to 120 min). The permeated drug was quantified by using the calibration curve obtained in concentrations range from 2.8 to 22.4 mg/L (*y* = 0.0559*x* + 0.051 *R*^2^ = 1). All experiments were performed in triplicate. As comparison, in vitro permeation test of raw CPA was carried out.

From the values obtained, the curves of accumulated amounts (mg/cm^2^) versus permeation time (min) were drawn; then, the flux (J) was determined from the slope of the steady state portion as well as the lag time (T lag) values from the x-intercept of the slope at a steady state [27].

### 2.4. In Vivo Studies

#### 2.4.1. In Vivo CPA Administration and Quantification

Male Wistar rats (200−250 g) anesthetized during the experimental period received a femoral intravenous infusion of 0.1 mg/mL CPA dissolved in a medium constituted by 20% (*v*/*v*) DMSO and 80% (*v*/*v*) physiologic solution, with a rate of 0.2 mL/min for 10 min. At the end of infusion and at fixed time points, blood samples (100 μL) were collected and cerebrospinal fluid (CSF) samples (50 μL) were withdrawn by the cysternal puncture method described by van den Berg and co-workers [28], which requires a single needle stick and allows the collection of serial (40–50 μL) CSF samples that are virtually blood-free [29]. Four rats were employed for femoral intravenous infusions. CSF samples (10 μL) were immediately injected into high performance liquid chromatography (HPLC) system for CPA detection. 

The blood samples were hemolyzed immediately after their collection with 500 μL of ice-cold water, then 50 μL of 3N NaOH and 100 μL of internal standard (10 μM CHA) were added. The samples were extracted twice with 1 mL of water-saturated ethyl acetate, and, after centrifugation, the organic layer was reduced to dryness under a nitrogen stream. One hundred and fifty microliters of a mobile phase (see below) was added, and, after centrifugation, 10 μL was injected into the HPLC system for CPA detection. 

The in vivo half-life of CPA in the blood was calculated by nonlinear regression (exponential decay) of concentration values in the time range within 90 min after infusion.

Nasal administration of CPA was performed to anaesthetized rats laid on their backs, following two procedures. The first way consisted on the introduction in each nostril of rats 20 μL of an aqueous suspension of CPA (5 mg/mL) using a semiautomatic pipette which was attached to a short polyethylene tubing [29]. After the administration, blood (100 μL) and CSF samples (50 μL) were collected at fixed time points, and they were analyzed with the same procedures described above. Four rats were employed for nasal administration of CPA suspension. The second way consisted on the insufflation of loaded CPA microparticles to each nostril of the anaesthetized rats by means of single dose Monopowder P1 insufflators [30]. The insufflators were loaded with about 2.4 mg of CH100, MC100 and MC50 and their content was administered to each nostril of rats. After the administration, blood (100 μL) and CSF samples (50 μL) were collected at fixed time points, then analyzed. Four rats were employed for nasal administration of each type of microparticulate powders.

All in vivo experiments were performed in accordance with the European Communities Council Directive of September 2010 (2010/63/EU) a revision of the Directive 86/609/EEC and were approved by the Italian Ministry of Health and by the Ethical Committee of the University of Ferrara. Any effort has been done to reduce the number of the animals and their suffering.

The area under concentration curves of CPA in the blood and CSF (AUC, μg mL^−1^ min) were calculated by the trapezoidal method. All the calculations were performed by using the computer program Graph Pad Prism (GraphPad Software Incorporated, La Jolla, CA, USA).

#### 2.4.2. HPLC Analysis

The quantification of CPA in all samples generated from the experimental procedures was performed by HPLC. The chromatographic apparatus consisted of a modular system (model LC-10 AD VD pump and model SPD-10A VP variable wavelength UV−vis detector; Shimadzu, Kyoto, Japan) and an injection valve with 20 μL sample loop (model 7725; Rheodyne, IDEX, Torrance, CA, USA). Separations were performed at room temperature on a 5 μm Hypersil BDS C-18 column (150 mm × 4.6 mm i.d.; Alltech Italia Srl, Milan, Italy), equipped with a guard column packed with the same Hypersil material. Data acquisition and processing were accomplished with a personal computer using CLASS-VP Software, version 6.12 SP5 (Shimadzu Italia, Milan, Italy). The detector was set at 269 nm. The mobile phase consisted of a ternary mixture of acetonitrile, methanol and 10 mM acetate buffer (pH 4) with a ratio of 4/40/56 (*v*/*v*/*v*). The flow rate was 0.8 mL/min and the retention time of CPA and CHA were 4.1 and 5.8 min, respectively. 

The chromatographic precision, represented by relative standard deviations (RSD), was evaluated by repeated analysis (*n* = 6) of the same sample solution containing each of the examined compounds at a concentration of 10 μM. The solutes were diluted in water by 10^−2^ M stock solutions in DMSO. The RSD values ranged between 0.81% and 0.83% for the analyzed compounds. The calibration curve of peak areas versus concentration was generated in the range 0.1 to 20 μM of CPA and resulted linear (*n* = 9, *r* = 0.998, *p* < 0.0001). For CSF simulation, standard aliquots of balanced solution (PBS Dulbecco’s without calcium and magnesium) in the presence of 0.45 mg/mL BSA were employed [31,32]. In this case, the chromatographic precision was evaluated by repeated analysis (*n* = 6) of the same sample solution containing 1.0 μM CPA whose RSD value was 0.92% and calibration curve of peak areas versus concentration was generated in the range 0.08 to 10 μM (corresponding to 26.8 to 3353 ng/mL), resulting linear (*n* = 8, *r* = 0.994, *p* < 0.0001). The LOD and LOQ values of CPA determination in the blood were 8.12 ng/mL (0.0002 nmoles/injection, signal-to-nose ratio of 3:1) and 26.8 ng/mL (0.0008 nmoles/injection, signal-to-noise ratio of 10:1), respectively. The accuracy of the analytical method for CPA extracted from rat whole blood was determined by comparing the peak areas of 10 μM CPA (corresponding to 3353 ng/mL) extracted at 4 °C (*n* = 3) with those obtained by injection of an equivalent concentration of the analyte dissolved in the mobile phase for HPLC analysis. The average recovery from rat whole blood ± S.E. was 86 ± 3%. The concentrations of CPA were therefore referred to as peak area ratio with respect to the internal standard CHA. The precision of this peak area ratio-based method is demonstrated by the RSD values of 1.03% for 10 μM CPA extracted from rat blood at 4 °C, whose calibration curve was linear over the range 0.15−30 μM (corresponding to 50.4 to 10,059 ng/mL; *n* = 9, *r* > 0.992, *p* < 0.0001). The LOD and LOQ values of CPA determination in the blood were 15.3 ng/mL (0.0005 nmoles/injection, signal-to-nose ratio of 3:1) and 50.4 ng/mL (0.0015 nmoles/injection, signal-to-noise ratio of 10:1), respectively.

### 2.5. Statistical Analysis

Data were analyzed using one-way ANOVA followed by Tukey or Bonferroni test (GraphPad Prism, version 6.02; GraphPad Software Incorporated). Difference was considered statistically significant at P values less than 0.05.

## 3. Results and Discussion

### 3.1. Preparation and Characterization of Microspheres

Spray drying appears to be a suitable method for the preparation of CPA-loaded microspheres containing methyl-β-cyclodextrin and chitosan chloride. The technique is simple and rapid as it only requires the preparation of a feed solution. Production yields were good (range from 63–72% (*w*/*w*)) with no significant differences between the formulations produced.

#### 3.1.1. Determination of Drug Content and Encapsulation Efficiency 

CPA content of the formulations and encapsulation efficiency are listed in Table 1. The drug contents of the formulations were relatively close to the theoretical values, and the encapsulation efficiency values ranged between 80% and 88%. The higher chitosan content of MC10 determined a significant decrease in drug encapsulation capacity compared to the other formulations (*p* < 0.05). 

#### 3.1.2. Morphological Analysis

**Figure 1:**
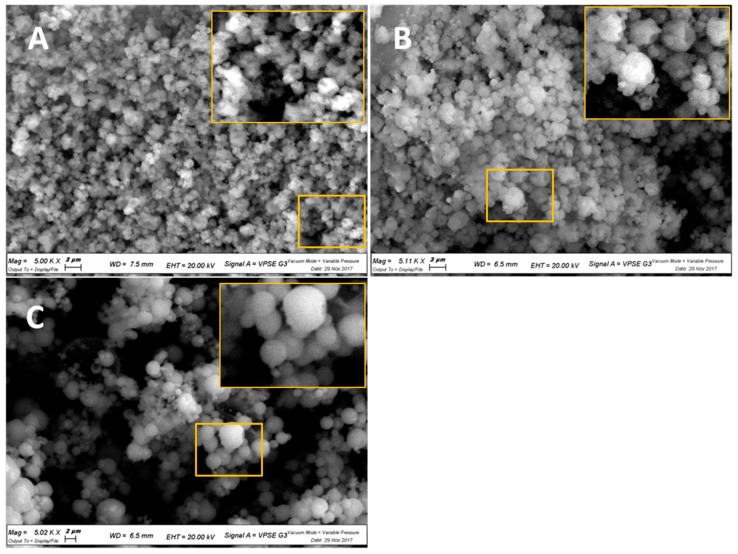
SEM micrographs with magnifications of 5000 times of: (**A**) MC90; (**B**) MC50; (**C**) MC10.

**Figure 2:**
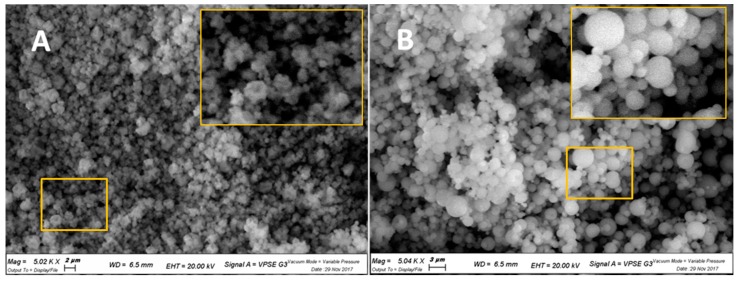
SEM micrographs with magnifications of 5000 times of: (**A**) MC100; (**B**) CH100.

Figure 1 illustrates that by increasing the percentage of methyl-β-cyclodextrin in the microspheres, the transition from a smooth to corrugated surface was gradual. In fact, CH100 and MC100 evidenced distinct morphologies. In particular, as reported in Figure 2, CH100 showed a spherical shape and smooth surface, whereas the surface of MC100 particles appeared corrugated in the presence of multiple invaginations.

#### 3.1.3. Particle Size Analysis

The d_vs_ values of the formulations are reported in Table 1. The size of the microspheres, generated by spraying feed solutions with the same concentration, depends on the kind and ratio of excipients used. In fact, the weight ratio between two polymers affected the d_vs_, which increased significantly from MC90 to MC50 and 10–90 (*p* < 0.05), but not particle size distribution (*p* > 0.05). In fact, SPAN index values were comparable, ranging between 1.85 and 2.24, indicating an almost narrow size distribution in all cases. Drug encapsulation did not significantly affect the particle size of methyl-β-cyclodextrin microspheres (MCb 4.52 ± 0.09). On the contrary, chitosan unloaded microparticles generally appeared bigger than the corresponding loaded particles. These last data would appear to be in accordance with those obtained in previous studies: indeed, size modification was observed for spray-dried chitosan microparticles as a consequence of the presence of metoclopramide [33] and CPA [23].

#### 3.1.4. Powder X-ray Diffraction

**Figure 3:**
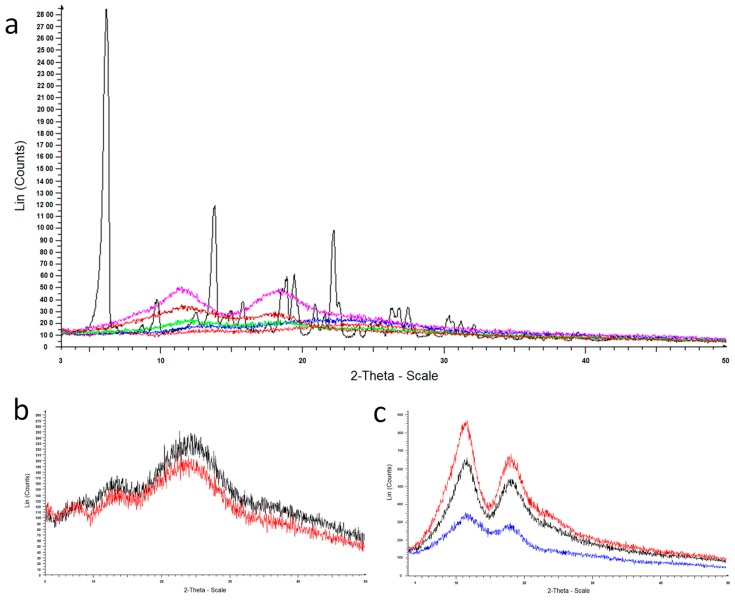
Powder X-ray diffraction patterns of: (**a**) CPA (black), MC90 (brown), MC50 (green) MC10 (blue), MC100 (fuchsia) and CH100 (red); (**b**) chitosan (black) and CH100 (red); (**c**) methyl-β-cyclodextrin (red), MCb (black) and MC100 (blue).

Additional information on the solid state of the microparticles was obtained by powder X-ray diffraction. As illustrated in Figure 3A, pure CPA (black) exhibited distinct diffraction peaks. The diffractogram of the loaded microparticles did not exhibit any CPA diffraction peaks, indicating the absence of its crystalline state in the loaded form. In particular, the patterns of CH100 (red) and MC10 (blue) samples reported in Figure 3A are accordant with the pattern of chitosan, as shown in Figure 3B, showing a magnification of the comparison between pure chitosan (black) and the sample CH100 (red) diffractograms. Moreover, the patterns of MC100 (fuchsia) and MC90 (brown) samples reported in Figure 3A are accordant with the pattern of methyl-β-cyclodextrin, as may be seen in Figure 3C, showing a magnification of the comparison among pure methyl-β-cyclodextrin (red), MCb (black) and MC100 (blue) diffractograms. The pattern of MC50 sample (green) reported in Figure 3A evidenced the presence of both chitosan and methyl-β-cyclodextrin in the same sample.

#### 3.1.5. Water Uptake 

**Figure 4:**
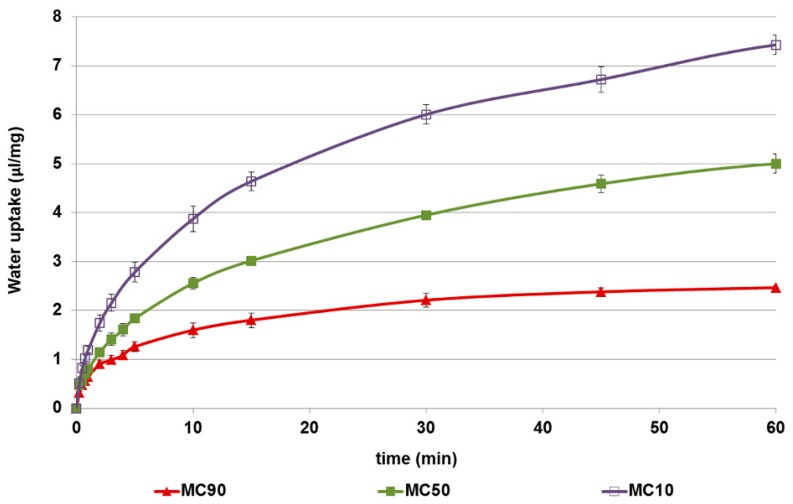
The water uptake capacity (μL/mg) of the microspheres. Data are reported as the mean value of three independent experiments. SD values were ≤0.17 of their corresponding means.

The water uptake of formulations containing both excipients was affected by their weight ratio (Figure 4). It is well-known that methyl-β-cyclodextrin is a water soluble excipient that absorbs very little water and is then rapidly solubilized; on the contrary, chitosan salts are able to absorb water allowing them to swell and gel [20]. Consequently, the amount of water absorbed per milligram of microspheres markedly increased when the amount of chitosan was raised in the particle matrix.

#### 3.1.6. In Vitro Permeation Test of Microspheres

**Figure 5:**
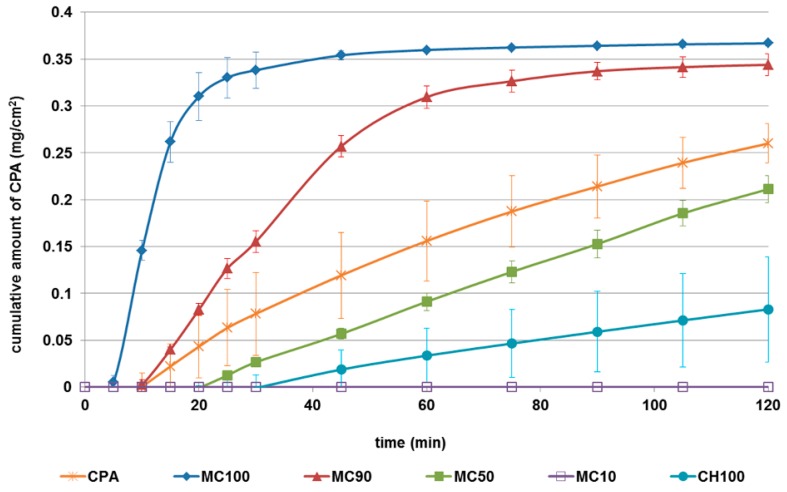
The permeation profiles of N6-cyclopentyladenosine (CPA) from formulations through lipophilic membranes. Profiles are compared with those of the raw drug. Data are reported as the mean ± standard deviation (SD) of three independent experiments.

The in vitro permeation profiles of CPA released from microspheres, compared with those of the pure drug, are shown in Figure 5. CPA, as a hydrophilic drug, was able to permeate synthetic membrane with linear kinetic (*n*= 11, *r* = 0.984, *p* < 0.0001), reaching 80% of the amount tested in acceptor medium within 120 min. When encapsulated in microspheres the CPA permeation ability was modified in relation to the polymer and ratio employed as the polymeric matrix. In particular, cyclodextrin increased the flux and total amount of drug permeated when used alone (MC100), or at 90% (*w*/*w*) (MC90) (Table 2). 

On the contrary, chitosan reduced the permeation of the loaded drug through lipophilic membranes (CH100: 30% of CPA permeated after 120 min). When the concentration of chitosan was 50% (MC50) and 90% (MC10) MC50, the CPA permeation flux and drug total amount significantly decreased (Table 2), reaching the zero values for MC10 sample at all time points tested (Figure 5). CPA’s inability to permeate when encapsulated in MC10 could be due to the need of the elevated amount of water for its hydration (as demonstrated by the water uptake results), which was not available in the experimental conditions. Worthy of note is, firstly, chitosan’s ability to reduce the permeation, and, secondly, methyl-β-cyclodextrin’s ability to increase the passage of the loaded drug through lipophilic membranes regardless of the kind of drug encapsulated [20]. Among the combined formulations tested, of interest is the linear kinetic of CPA release from MC50 in the range from 20 min to 120 min (R^2^ = 0.9987). 

Lag time is the time required for the diffusion flow to become stable. During our experiments, it varied from 1.44 to 20.11 min (Table 2), and was affected by the hydration time of the matrix. These lag times could be considered appropriate for nasal formulations, taking into account that the half-life of clearance in nasal cavity is in the order of 15–20 min [7].

On the basis of the in vitro properties of microspheres made by combined penetration enhancers, MC50 was elected as the leader formulation to be in vivo tested. MC50 possessed good EE% as well as size and morphology, comparable to the other formulation containing a high amount of chitosan (MC10). Nevertheless, MC50 needed lower amounts of water to hydrate and showed a better drug release profile than MC10. On the other hand, MC90 had very similar in vitro properties to MC100, but as it is a formulation made by cyclodextrin alone, it would not be possible to evaluate the in vivo effect of the co-presence of the two penetration enhancers.

### 3.2. In Vivo CPA Administration and Quantification

The analysis of rat blood samples following the intravenous infusion of 0.2 mg of CPA indicated that the drug concentration in the blood stream was 6.11 ± 0.41 μg/mL at the end of infusion, and decreased with a terminal half-life of 6.8 ± 0.9 min. The AUC value of this profile was 37687 ± 1118 ng mL^−1^ min (Table 3). These data are in good agreement with those obtained in previous in vivo studies on CPA pharmacokinetics in rats [23,34]. On the other hand, no CPA was detected in the CSF of rats within 120 min after the end of the intravenous infusion. These results confirm that CPA is unable to reach the CNS from the bloodstream, as previously reported [23,35,36,37]. The microparticles (CH100, MC100 and MC50) were tested for nasal administration of CPA in order to verify its potential uptake into the CNS. Nasal administration of an aqueous suspension of CPA was also tested as control. Indeed, the nasal administration of pure CPA as powder was not performed, owing to the very low dose (200 μg in rats). Therefore, as control we employed a water suspension of the raw drug. No significant amounts of CPA were observed either in blood or in in the CSF of rats within 120 of the treatment (data not shown). A similar behavior was registered after the nasal administrations of rokitamicin [15], deferoxamine [20], and a zidovudine prodrug [16,29], as water suspensions, confirming that appropriate nasal formulations are needed in order to promote the drug uptake in the central nervous system.

**Figure 6:**
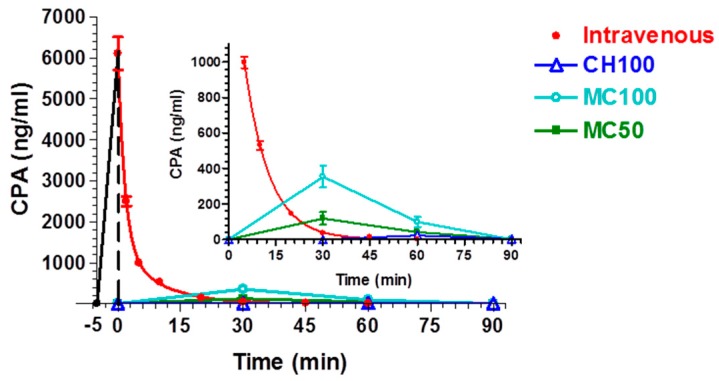
Blood CPA concentrations (ng/mL) after infusion or nasal administration of a 200-μg dose. The time ranging from −5 to 0 min refers to CPA IV infusion. In the inset: zoom of the same plot with expanded Y-scale (CPA blood concentration) from 0 to 1000 ng/mL. The inset highlights the concentration values obtained after administration of CH100, MC100 and MC50. Data are expressed as the mean ± standard deviation, *n* = 4.

**Figure 7:**
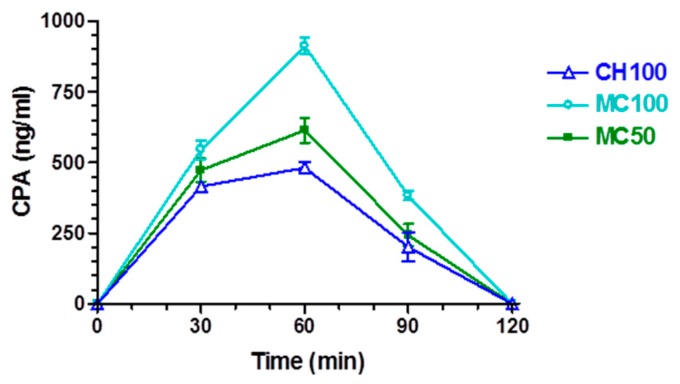
CPA concentrations (ng/mL) detected in the cerebrospinal fluid (CSF) after nasal administration of CH100, MC100 and MC50. Data are expressed as the mean ± standard deviation, *n* = 4.

On the contrary, nasal administration of the powder constituted by the loaded CH100, MC100 and MC50 microparticles (4.8 mg, about 200 μg of CPA) produced detectable levels of the drug in both blood and CSF, as reported in Figure 6 and Figure 7, respectively. CH100 microparticles were previously described [23], but they were chosen as reference formulation in order to evaluate the effects induced by MC100 presence in the formulations. After nasal administration of CH100 particles, the peak concentration in the blood was detected at 60 min, with values just above the LOD of the analytical method (22 ± 12 ng/mL), indicating relatively poor CPA permeation from the nose to the bloodstream, as previously reported for this type of formulation [23]. Indeed, a comparison of the AUC values (Table 3) obtained after intravenous administration (37687 ± 1118 ng mL^−1^ min) and nasal administration (699 ± 257 ng mL^−1^ min) indicated an absolute bioavailability value of 1.85% for the microparticulate powders based on chitosan. 

The presence of cyclodextrins in the microparticulate nasal formulation induced an increase in CPA permeation in the bloodstream after nasal administration. Indeed, as evidenced in the inset of Figure 6, following the nasal administration of MC50 or MC100 particles, the peak plasma concentrations of CPA were detected at 30 min, with values of 118 ± 36 ng/mL or 354 ± 62 ng/mL, respectively. In this case the AUC values of MC50 or MC100 formulations were 4830 ± 827 or 13,590 ± 1451 ng mL^−1^ min, respectively, but only the MC100 value appeared significantly different from the AUC of CH100 (Table 3). The absolute bioavailability values were 12.8% and 36.0% for MC50 and MC100 samples, respectively. Table 3 summarizes the AUC and bioavailability values for the different administration routes of CPA. These data indicate that the progressive modifications of the morphology and structural composition of CH100 particles induced by the enhancement of the CDs in their composition reflects a progressive increase in systemic delivery of CPA, following their nasal administration. 

Relevant amounts of CPA were detected in the CNS of rats after nasal administration of the solid microparticulate formulations. In particular, Figure 7 shows the CPA concentrations detected in the CSF: drug loaded chitosan microparticles (CH100 sample) may be seen to lead to an increase in CPA concentration values up to 481 ± 21 ng/mL within 60 min. Then, at 90 min the CPA concentration in CSF was significantly decreased (201 ± 50 ng/mL) and became undetectable at 120 min. The AUC value of this profile was found to be 32,910 ± 1199 ng mL^−1^ min. These data are in good agreement with those obtained in previous in vivo studies for this type of microparticles [23]. As registered in the bloodstream, the presence of cyclodextrins in the nasal formulations induced an increase in CPA amounts permeating in the CSF. Indeed, after nasal administration of the samples MC50 or MC100, the CPA concentrations in the CSF of rats increased up to 614 ± 44 and 911 ± 29 ng/mL, respectively, within 60 min after the nasal administration, then the values decreased to 243 ± 39 ng/mL for MC50 and 382 ± 16 ng/mL for MC100 at 90 min and became undetectable at 120 min. The AUC value of the MC50 and MC100 profiles were found to be 39,900 ± 1520 and 55,140 ± 1016 ng mL^−1^ min, respectively, with relative bioavailability values of 121% for MC50 and 170% for MC100, with respect to the CH100 formulation. These data seem to confirm that the presence of cyclodextrins in microparticulate nasal formulations can enhance drug permeation across nasal mucosa, with respect to formulations based on chitosan [20].

**Figure 8:**
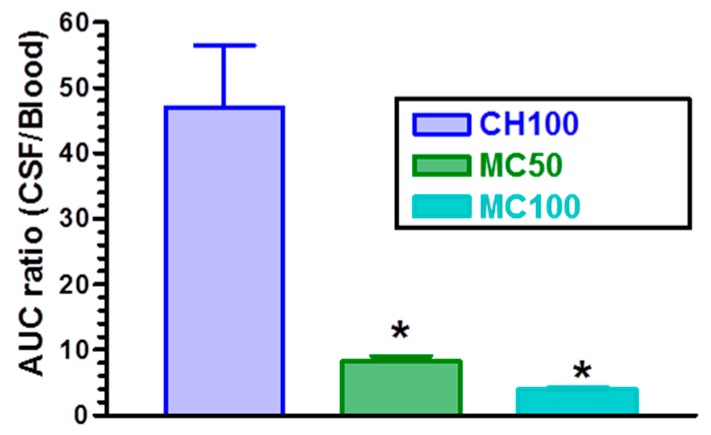
Ratios between CPA area under concentration curves (AUC) values between CSF and bloodstream obtained after nasal administration of the loaded microparticles CH100, MC50 and MC100. Data are expressed as the mean ± standard deviation, *n* = 4. * *p* < 0.001 versus CH100.

On the other hand, CH100 microparticles allowed us to obtain a CPA distribution between CSF and bloodstream resulting in a significantly higher ratio of concentrations than the ratios obtained by the nasal administration of the microparticles containing cyclodextrins. This aspect is evidenced in Figure 8, where the AUC ratios of CPA between CSF and bloodstream, obtained after nasal administration of the CH100, MC50 and MC100 microparticles, are reported. In particular, CH100 microparticles induced an AUC ratio of 47 ± 18, significantly higher (*p* < 0.001) than the AUC ratios obtained by nasal administration of MC50 (8.2 ± 1.6) and MC100 (3.9 ± 0.5) samples. A high AUC value can be very useful when a selective therapeutic activity of the drug is required in the central nervous system with respect to the bloodstream where the drug activity can induce severe side-effects. To the best of our knowledge, this is the first time that a modulation between the optimal uptake of a drug and its optimal distribution between CSF and bloodstream has been shown to be easily obtainable simply by varying the amount of cyclodextrins and chitosan in the microparticles.

## 4. Conclusions

This study has demonstrated that the choice of penetration enhancer in nasal microspheres significantly affects the in vivo bioavailability of the encapsulated drug. In particular, methyl-β-cyclodextrin is more effective in enhancing drug permeation through respiratory and olfactory epithelia; this could be due to the changes in both paracellular and transcellular transports. On the contrary, chitosan has proven to be a selective excipient able to increase, above all, the paracellular transport of a drug through olfactory epithelia. This is, moreover, the first time that optimal drug distribution between CSF and bloodstream has been shown to be easily obtainable by varying the amount of these two penetration enhancers studied in the matrix of powder formulations.

## Figures and Tables

**Table 1:** The polymer composition (% *w*/*w*), drug content (DC) and encapsulation efficiency (EE) of loaded microspheres.

Microsphere Codes	Methyl-β-Cyclodextrin	Chitosan	DC (%)	EE (%)	d_vs_ (µm)	SPAN
MC100	100	-	4.25 ± 0.05 ^a^	88.78 ± 0.98 ^a^	3.49 ± 0.10	2.20
MC90	90	10	4.25 ± 0.01 ^a^	88.66 ± 0.22 ^a^	2.74 ± 0.09	1.85
MC50	50	50	4.20 ± 0.07 ^a^	87.87 ± 1.53 ^a^	6.79 ± 1.84 ^b,c^	2.24
MC10	10	90	3.81 ± 0.05	80.91 ± 1.13	6.55 ± 1.04 ^b,c^	2.09
CH100 ^#^	-	100	4.75 ± 0.06	95.0 ± 1.20	6.14 ± 0.84 ^b,c^	1.66

^#^ Prepared as reference for in vivo studies [23]. ^a^ *p* < 0.05 MC10 versus MC100; versus MC90; versus MC50. ^b^ *p* < 0.05 MC100 versus CH100; versus MC50; versus MC10. ^c^ *p* < 0.05 MC90 versus CH100; versus MC50; versus MC10.

**Table 2:** Values of flux (J), T lag and drug permeated for each formulation after 120 min.

Microsphere Codes	J (μg/cm^2^ min)	T Lag (min)	Drug Permeated (%)
CPA	2.48 ± 0.65 ^a,b,c^	1.44	83.55 ± 10.85 ^d,e,g^
**MC100**	25.64 ± 2.14 ^a^	4.64	112.56 ± 1.24 ^d^
**MC90**	7.27 ± 0.18 ^a,b^	8.96	105.87 ± 2.26 ^e^
**MC50**	2.09 ± 0.14 ^a,b^	17.62	65.46 ± 3.66 ^d,e,f^
**MC10**	0.00 ^a,b,c^	-	0.00
**CH100**	0.86 ± 0.53 ^a,b^	20.11	22.26 ± 15.33 ^d,e,f,g^

^a^*p* < 0.01 MC100 versus MC90; versus MC50; versus MC10; versus CH100 and CPA. ^b^ *p* < 0.01 MC90 versus MC50; versus MC10; versus CH100; versus CPA. ^c^ *p* < 0.05 MC10 versus CPA. ^d^ *p* < 0.05 MC100 versus MC50; versus CH100; versus CPA. ^e^ *p* < 0.05 MC90 versus MC50; versus CH100; CPA. ^f^ *p* < 0.05 MC50 versus CH100. ^g^ *p* < 0.05 CH100 versus CPA.

**Table 3:** Area under concentration curves (AUC) obtained after CPA administration in rats for blood and CSF compartments.

	Blood		CSF	
Administration Way	AUC (ng mL^−1^ min)	Absolute Bioavailability	AUC (ng mL^−1^ min)	Relative Bioavailability
Intravenous	37,687 ± 1118	100	n.d.	0
Nasal (suspension)	n.d.	0	n.d.	0
Nasal (CH100)	699 ± 257	1.85%	32,910 ± 1199	100%
Nasal (MC50)	4830 ± 827 ^a^	12.8%	39,900 ± 1520	121% ^b^
Nasal (MC100)	13,590 ± 1451 ^c^	36.0%	55,140 ± 1016	170% ^c^

Data are reported as mean ± standard deviation, *n* = 4. Absolute bioavailability values are referred to AUC obtained by intravenous administration (100%); relative bioavailability values are referred to AUC obtained by nasal administration of CH100 microparticles (100%). n.d., not detectable. ^a^ *p* > 0.05 versus CH100; ^b^ *p* < 0.05 versus CH100; ^c^ *p* < 0.001 versus CH100.
